# Improved Method for Obtaining Patient‐Derived Glioblastoma Organoid and Primary Cells: A Generic and Simplified Approach for Intracranial Tumor With Minimized Input

**DOI:** 10.1002/cns.71024

**Published:** 2026-09-29

**Authors:** Siyuan Wang, Zhonggang Shi, Chihao Zhu, Dawei Dai, Xi Yang, Zhiyi Zhou, Qianying Qiu, Wenhua Sun, Yifeng Miao, Yongming Qiu

**Affiliations:** ^1^ Department of Neurosurgery Renji Hospital, Shanghai Jiao Tong University School of Medicine Shanghai China; ^2^ Shanghai Institute of Thoracic Oncology Shanghai Chest Hospital, Shanghai Jiao Tong School of Medicine Shanghai China; ^3^ State Key Laboratory of Cell Biology, Shanghai Institute of Biochemistry and Cell Biology, Center for Excellence in Molecular Cell Science Chinese Academy of Sciences, University of Chinese Academy of Sciences Shanghai China; ^4^ Department of Otorhinolaryngology‐Head & Neck Surgery Xinhua Hospital, Shanghai Jiao Tong University School of Medicine Shanghai China; ^5^ Department of Neurosurgery Pujiang Hospital Shanghai China

**Keywords:** cancer modeling, drug testing, glioblastoma, intracranial tumors, organoid, primary tumor cell

## Abstract

**Introduction:**

Glioblastoma is one of the most malignant primary tumors in adults. The shortages of the traditional tumor models obstruct the development of basic research and therapeutic modes. Application of organoid is critical to keep tumoral heterogeneity and microenvironment. The current method for generating glioblastoma organoid is sophisticated, laborious, and inconvenient.

**Methods:**

We collected glioblastoma tissue samples from patients and used an improved methodology to generate organoids. Subsequently, the collected suspension during the process was used to isolate primary tumor cells. Organoid were used to assess its drug susceptibility. Finally, we generated organoids of other CNS tumors with the same method.

**Results:**

The improved method took less time and lowered technical requirements. Glioblastoma organoids generated with our improved method can be cultured for further research. Primary glioblastoma cells isolated from collected medium were proved with immunofluorescence. Patient‐derived glioblastoma organoids showed consistent drug susceptibility with the patients. Tumor organoids were also generated successfully from patient's lymphoma and low‐grade glioma samples.

**Conclusions:**

We improved an easier and rapider method to generate glioma organoids from tissue samples and avoided wasting primary tumor cells in used medium. This method established a technical foundation for the widely application of organoids in neuro‐oncology tumor research and clinical decision‐making.

## Graphical abstract

An improved and simplified method enables the generation of primary tumor cells and tumoroids from the same tissue sample with minimal input, applicable universally to both intracranial and spinal tumors. Comparative analysis of drug screening results with patient follow‐up data allows for the assessment of individual drug sensitivity.

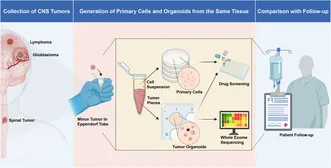

## 1 Introduction

Glioblastoma is widely known as the most common malignant cancer in the adult central nervous system (CNS) [1] with aggressive and invasive characteristics, and patients always have poor prognosis [2, 3]. Maximal surgical resection followed by radiation therapy and chemotherapy has been the current standard of clinical therapy for the past years. Despite numerous preclinical attempts, few treatments have demonstrated consistent efficacy in clinical trials [4]. Few patients with glioblastoma could be alive for more than 5 years after diagnosis [2]. Clinical decisions regarding postoperative adjuvant therapy were determined by tumor genotype profiling at present [5]. However, interpatient heterogeneity frequently leads to heterogeneous therapeutic responses to radiotherapy and chemotherapy, resulting in disease recurrence and metastatic progression despite multimodal interventions [6]. Even though there are many attempts based on proved experiments, few treatments received consistent results in clinical research. The inherent limitations of traditional models used in preclinical research, including cell lines [7, 8, 9] and animal models [10, 11, 12], may contribute to the failure of clinical translation. Establishing a new method to assist in formulating therapy for patients is important to improve the prognosis of the patients with glioblastoma, as well as neoplasms in the nervous system.

Recently, in vitro organoid models have been developed to stimulate the native structure and heterogeneity of various organs [13], such as cerebral organoid [14] with reliable electrical activity and neuronal network [15, 16]. Furthermore, organoids have been demonstrated to be significant for many other tumors including pancreas, lung, and liver cancers [17], and are widely applied in pharmacological development and mechanistic research [18, 19]. The GB‐like organoids were initially obtained as a gene‐edited cerebral organoid [12] to observe the occurrence of glioma [20]. Afterwards, a new model named GLICO was reported to generate organoids through co‐culture of cerebral organoid and glioblastoma stem cells (GSCs) [21]. Up to now, most researchers generate tumor organoids, including glioblastoma, by culturing tumor stem cells isolated from patient tissue samples in Matrigel with exogenous growth factors to form a three‐dimension (3D) structure similar to the derived tumor [22, 23, 24]. However, current organoid models exhibit incomplete preservation of the Tumor Microenvironment (TME), with selective depletion of critical stromal and cell populations during the experimental process inherent in organoid establishment. This limitation compromises the model's capacity to fully recapitulate the complex cellular interactions and physiological fidelity observed in native tumor ecosystems [25]. Subsequent co‐culture cannot maintain the heterogeneous composition of cells in the microenvironment from different tissues [26], which has been recognized as an important factor in glioblastoma development [27, 28, 29]. Therefore, Jacob et al. reported a method to generate glioblastoma organoids (GBO) from tumor pieces without using Matrigel [30]. The reported method enabled generation of glioblastoma organoids (GBOs) that maintain the microenvironment of glioblastoma, including astrocytes, microglial cells, and even the capillaries. Whereas, researchers need to use a stereomicroscope and dissection scissors to mince resected tumors into tiny pieces. This method cannot be performed to generate organoids for basic research unless new devices are equipped in their laminar flow biosafety cabinet. Practical application also shows that this method needs high technical requirements and takes a long time to mince small pieces in a glass dish with medium, which limits the volume of tumor tissues treated. Therefore, an improved method, which can be proceeded with simple steps, common devices, and widely used in different areas, is needed to popularize glioblastoma organoids as a better in vitro model in basic research and translational research in more labs.

Current researches on adult CNS tumors focus on glioblastoma. However, there are many other types of CNS tumors, such as low‐grade glioma, lymphoma, hypophysoma, and metastatic tumor [31], still lacking better in vitro models to stimulate their native characteristics. Cell lines and animal models have different defects in studying the mechanisms of tumor occurrence, development, microenvironment, and novel therapies of tumors [32]. Establishing a method to generate organoids of other cranial tumors will promote the development of basic research.

Here, we report an improved method to generate and culture GBOs. Compared with the original method, the improved method lessens time and basic equipment and is easier in operation. It also provided a new step to get patient‐derived primary glioblastoma cells at the same time. Additionally, we used glioblastoma organoids from different patients to test their susceptibility to temozolomide and performed comparative analysis with patients' clinical prognosis. Our work reveals a possibility to provide individual treatment based on the results of in vitro experiments. Finally, we generated organoids from low‐grade glioma and lymphoma with the same method successfully, which shows the potential of a universal method in generating organoid models from different kinds of CNS tumor samples.

## 2 Methods

### 2.1 Collection of Patient Tumor Samples

Patient glioblastoma tissues were collected from the department of neurosurgery, Shanghai Jiao Tong University School of Medicine Affiliated Renji Hospital since 2023. Twenty‐two cases of CNS primary tumor were surgically resected and confirmed a preliminary diagnosis according to the WHO pathological classification by the department of pathology, including glioblastoma (18 cases), subependymal tumor (1 case), anaplastic ependymoma (1 case), gliosarcoma (1 case), and diffuse large B‐cell lymphoma (1 case). Patient personal information, region of tumor, necrosis, vascular proliferation, and pathological results are shown in Tables 1 and 2.

**TABLE 1:** The information about the tumor samples from the patients.

No.	Patient	Age	Gender	WHO grade	Diagnosis	Region	Necrosis	VP
01	G 230417	68	F	IV	Glioblastoma	LF	Y	N
02	G 230422	69	F	III	Anaplastic ependymoma	LF	N	N
03	G 230626	60	M	IV	Glioblastoma	LF	N	N
04	G 230629	64	F	IV	Gliosarcoma	RT	Y	N
05	G 230727	68	F	IV	Glioblastoma	LO	Y	N
06	G 230728	71	F	IV	Glioblastoma	LT	N	N
07	G 230921	50	M	IV	Glioblastoma	RT	N	N
08	G 231021	64	F	IV	Glioblastoma	RO	N	N
09	G 240408	43	M	IV	Glioblastoma	LF	N	N
10	G 240425	76	F	IV	Glioblastoma	LP	N	N
11	G 240513	57	M	IV	Glioblastoma	RF	N	N
12	G 240514	69	M	IV	Glioblastoma (small cell)	CC	Y	N
13	G 240524	54	M	IV	Glioblastoma	BG	Y	N
14	G 240528	65	M	IV	Glioblastoma (recurrence)	LP	Y	N
15	G 240612	63	F	IV	Glioblastoma	RT	N	N
16	G 240926	67	F	IV	Glioblastoma	LF	Y	Y
17	G 241012	72	F	IV	Glioblastoma (giant cell)	RT	N	N
18	G 241028	38	M	IV	Glioblastoma	LP	N	N
19	G 241031	52	M	IV	Glioblastoma	RF	Y	Y
20	G 241125	38	M	/	Subependymoma	CSC	N	N
21	G 241128	55	M	IV	Glioblastoma	RF	Y	Y
22	G 241129	76	F	/	Diffuse large B‐cell lymphoma	LF	N	N

Abbreviations: BG, basal ganglia; CC, corpus callosum; CSC, cervical spinal cord; F, female; LF, left frontal; LO, left occipital; LP, left parietal; LT, left temporal; M, male; N, no; RF, right frontal; RO, right occipital; RT, right temporal; VP, vascular proliferation; Y, yes.

**TABLE 2:** Expression of the genes relating to prognosis of the patients.

No.	IDH1/IDH2		TERT		MGMT status	BRAF (V600E)	EGFR	Loss of heterozygosity				ATRX	GFAP	Ki67	Olig2	P53
	R132	R172	C228T	C250T				CDKN2A	10q23	1p36	19q13					
01	WT	WT	WT	MT	Non‐methylation	WT	N/A	Integrity	N/A	Deletion	Integrity	MT	MT	15%	MT	WT
02	WT	WT	MT	WT	Methylation	WT	WT	Integrity	Deletion	N/A	N/A	MT	MT	5%	MT	WT
03	WT	WT	WT	WT	Methylation	WT	WT	Integrity	Integrity	Integrity	Integrity	MT	MT	40%	MT	80%
04	WT	WT	MT	WT	Non‐methylation	WT	WT	Integrity	Integrity	Integrity	Integrity	MT	MT	30%	MT	90%
05	WT	WT	WT	MT	Methylation	WT	MT	Integrity	Integrity	Integrity	Integrity	N/A	MT	N/A	MT	N/A
06	WT	WT	MT	WT	Non‐methylation	WT	MT	Integrity	Deletion	Integrity	Integrity	MT	MT	15%	MT	N/A
07	WT	WT	MT	WT	Non‐methylation	WT	MT	Integrity	Integrity	Integrity	Integrity	MT	MT	20%	MT	60%
08	WT	WT	WT	MT	Non‐methylation	WT	WT	Deletion	Deletion	Integrity	Integrity	MT	MT	20%	MT	WT
09	WT	WT	WT	WT	Non‐methylation	WT	WT	Integrity	Integrity	Integrity	Integrity	MT	MT	30%	MT	70%
10	WT	WT	MT	WT	Methylation	WT	MT	Integrity	Deletion	Integrity	Integrity	MT	MT	80%	MT	95%
11	WT	WT	MT	WT	Non‐methylation	WT	MT	Integrity	Deletion	Integrity	Deletion	MT	MT	20%	MT	20%
12	WT	WT	MT	WT	Non‐methylation	WT	WT	Deletion	Integrity	Integrity	Integrity	MT	MT	70%	MT	90%
13	WT	WT	WT	MT	Non‐methylation	WT	MT	Deletion	Deletion	Integrity	Integrity	N/A	MT	20%	MT	WT
14	N/A	N/A	N/A	N/A	N/A	N/A	N/A	N/A	N/A	N/A	N/A	MT	WT	40%	WT	60%
15	WT	WT	WT	WT	Non‐methylation	WT	WT	Integrity	Integrity	Integrity	Integrity	MT	MT	60%	MT	WT
16	WT	WT	WT	MT	Non‐methylation	WT	WT	Integrity	Deletion	Integrity	Integrity	MT	MT	40%	MT	3%
17	WT	WT	WT	WT	Methylation	WT	WT	Integrity	Deletion	Integrity	Integrity	MT	MT	20%	N/A	35%
18	WT	WT	WT	WT	Non‐methylation	WT	WT	Integrity	Integrity	Integrity	Integrity	WT	MT	70%	MT	MT
19	WT	WT	MT	WT	Methylation	WT	WT	Integrity	Deletion	Integrity	Integrity	MT	MT	50%	MT	60%
20	N/A	N/A	N/A	N/A	N/A	N/A	N/A	N/A	N/A	N/A	N/A	N/A	MT	10%	MT	N/A
21	WT	WT	N/A	N/A	N/A	N/A	MT	Deletion	Deletion	Integrity	Deletion	MT	MT	70%	MT	80%
22	N/A	N/A	N/A	N/A	N/A	N/A	N/A	N/A	N/A	N/A	N/A	N/A	N/A			

Abbreviations: ATRX, a‐thalassemiamentalretardation syndrome X; BRAF, B‐Raf proto‐oncogene; CDKN2A, cyclin dependent kinase inhibitor 2A; EGFR, epidermal growth factor receptor; GFAP, glial fibrillary acidic protein; IDH, isocitrate dehydrogenase; MGMT, O‐6‐methylguanine‐DNA methyltransferase; MT, mutant type; N/A, the patient was not tested for genes; Olig2, oligodendrocyte transcription factor 2; TERT, telomerase reverse transcriptase; WT, wild type.

### 2.2 Follow‐Up of Glioblastoma Patients

The glioblastoma patients involved in our research were called for follow‐up. The information of overall survival (OS), progression‐free survival (PFS), the score of Karnofsky Performance Status (KPS), and treatments after operations was analyzed with the results of in vitro organoids experiments and shown in Table 3.

**TABLE 3:** The results of the patients' follow‐up.

No.	Patient	OS	PFS	KPS	Clinical treatment
01	G 230417	2023.09	—	—	—
02	G 230422	Lost	2023.11	—	—
03	G 230626	Alive	No progression	100	Temozolomide and radiotherapy
04	G 230629	2023.11	—	—	—
05	G 230727	Lost	—	—	Operation after recurrence
06	G 230728	2024.8.11	—	—	—
07	G 230921	Lost	—	—	—
08	G 231021	Alive	2024.7	30	—
09	G 240408	Alive	No progression	100	—
10	G 240425	Alive	2024.8	30	—
11	G 240513	Alive	No progression	100	—
12	G 240514	2024.8	—	—	—
13	G 240524	Alive	2024.7	40	—
14	G 240528	Alive	2024.6	30	—
15	G 240612	Alive	—	30	—
16	G 240926	Alive	—	40	—

Abbreviations: KPS, Karnofsky performance status; OS, overall survival; PFS, progression‐free survival.

### 2.3 Generation and Culture of GBOs From Resected Tumor Tissues

The tumor tissues were placed in preservation medium containing Hibernate A (Thermo Fisher Scientific, A12475‐01), 1× GlutaMax (Thermo Fisher Scientific, 35050‐061), and 1× Antibiotic‐Antimycotic (100×, Thermo Fisher Scientific, 15240‐096) and transferred into a petri dish with the same medium in a laminar flow biosafety cabinet within 1 h after collecting samples. Extended ex vivo intervals between tissue collection and processing induce progressive structural degradation of tissues, which is characterized by compromised cellular cohesion and extracellular matrix destabilization. The morphologic change reduced the success rate of organoid generation, ultimately undermining the experimental fidelity of patient‐derived tumor models. Blood vessels, normal tissues, and tissues treated with electrocoagulation were removed with scissors and tweezers. Blood and small tissue pieces were removed by preservation medium. Some tumor pieces were frozen and stored at −80°C for further sequencing. The other tumor tissues were dissected into 0.1 to 0.2‐mL pieces. Several pieces were transferred into a 1.5‐mL centrifuge tube (Biosharp, BS‐15‐M) with preservation medium and minced with dissection scissors until most of the tissues were minced into pieces with less than 1.0‐mm diameter. This step was repeated until all pieces were minced into small pieces and the tumor cells and tissue debris were removed with preservation medium. Preservation medium used to wash tumor pieces was collected in centrifuge tubes. The tumor pieces were incubated in 1× RBC lysis buffer (Thermo Fisher Scientific, 2968482) at room temperature for minutes to lyse red blood cells and washed with the preservation medium after aspirating RBC lysis buffer.

The remaining tumor pieces were distributed into non‐TC treated 6‐well culture plates (Nest, 041723BG010418B) with 4‐mL culture medium containing 50% DMEM/F12 (Thermo Fisher Scientific, C11330500BT), 50% Neurobasal‐A (Thermo Fisher Scientific, 10888‐022), 1× GlutaMax, 1× N2 supplement (Thermo Fisher Scientific, A13707‐01), 1× B27 supplement (Thermo Fisher Scientific, 12587‐010), 1× 2‐mercaptoethanol (Thermo Fisher Scientific, 21985‐023) and 2.5 mg/mL human insulin (MedChemExpress, HY‐P0035) per well. The organoids were cultured on a shaker rotating at 120 rpm in an incubator of 37°C, 5% CO_2_ and 90% humidity. Within the first few days of culture, the medium was cloudy, and nearly 75% of the medium in wells was replaced with fresh medium every 2 days to remove the dead cells and tissue debris. Approximately 1 week later, the edge of some tumor pieces became round and smooth, indicating the organoids' formation. The organoids with a smooth appearance were transferred into new plates for further culture. Remained pieces were kept culturing and observed. Many tumor pieces formed round organoids after 2 weeks. The successfully established GBOs were spherical, grew continuously, and kept growing.

The GBO had been cut into pieces with less than 500‐μm diameter to prevent limitation of nutrient and oxygen diffusion in the center when its diameter exceeded 1 mm. Treating the GBO with perpendicular cuts twice dissected it into 4 pieces. The treated pieces should be cut perpendicularly until all the GBOs are cut into pieces with a diameter of 300 to 500 μm. The treated GBO pieces should be selected in 6‐well culture plates for culturing and would form spherical GBOs with smooth edges in 3 to 5 days.

### 2.4 Isolation and Culture of Patient‐Derived Primary Tumor Cells

The preservation medium used to wash the tumor pieces during generation of GBOs was filtered twice with 70‐μm cell filters (Biosharp, 22311857) to remove tissue debris. The cell suspension was collected and centrifuged at 1000 rpm for 4 min, and the supernatant was aspirated. The cell pellet was resuspended and incubated with 1 to 2 mL 1× RBC lysis buffer at room temperature for 5 min and centrifuged at 1000 rpm for 4 min. This step was repeated until most red blood cells were lysed. The cell pellet was resuspended and transferred to a 25‐cm^2^ cell culture flask (Nest, 707001). After 48 to 72 h, the medium in the flask was aspirated, and the adherent cells were washed with PBS (Thermo Fisher Scientific, 10010023) to remove debris and dead cells. The primary cells were kept cultured with DMEM/F12 medium containing 2% B27 supplement and 1% GlutaMax.

### 2.5 Biobanking and Recovery of GBOs and Primary Cells

To store spare organoids, GBOs with more than 1.0‐mm diameter were minced into pieces with 100 to 200‐μm diameter and removed tissue debris with culture medium. The treated GBO pieces were incubated with culture medium containing 10 μM Y‐27632 (MedChemExpress, HY‐10071) for 1 h. The GBO pieces were resuspended in cryovials (LABSELECT, 22321029R) with culture medium containing 10% DMSO (Thermo Fisher Scientific, 85190) and 10 μM Y‐27632 at room temperature for 15 min. The treated GBOs were gradient‐cooled to −80°C and stored in a refrigerator at −80°C. For recovery, cryovials were thawed in a water bath at 37°C quickly and the stored GBO pieces were transferred in a tube to aspirate medium. The GBO pieces were resuspended and cultured in culture medium containing 10 μM Y‐27632, and the medium should be replaced with pure culture medium the next day. Survival of the GBOs was confirmed after 1 to 2 weeks and the dead GBOs were removed.

When the density of primary glioblastoma cells exceeded 80%, the medium was aspirated and the flasks were washed with PBS. The primary cells were digested with 0.25% trypsin (Thermo Fisher Scientific, 25200027) for 2 to 3 min, and ceased the digestion with DMEM/F12 medium containing 10% fetal bovine serum. The cell suspension was centrifuged at 1000 rpm for 4 min to aspirate medium, and the cell pellet was resuspended with 1 mL serum‐free cell freezing medium (NCM Biotech, C40100) in cryovials. The primary cells were gradient‐cooled to −80°C and stored at −80°C in a refrigerator for further experiments. For recovery, the cryovials were thawed quickly in a water bath at 37°C and the cell suspension was transferred into a 15‐mL tube with 4 mL DMEM/F12 medium containing 10% fetal bovine serum. The cell suspension was centrifuged at 1000 rpm for 4 min to aspirate the cell freezing medium. The cell pellet was resuspended with DMEM/F12 medium containing 2% B27 supplement and 1% GlutaMax and cultured in a 25‐cm^2^ cell culture flask.

### 2.6 Tissue Processing and H&E Staining

The parental tumor and GBOs were snap‐frozen in liquid nitrogen and sliced using a cryostat (Leica), and melted onto slides (Biosharp, BC014). The slides were dried for H&E staining at room temperature. Slides were treated using 4% paraformaldehyde (PFA) for 15 min at 4°C and washed with PBS. The slices were next stained with hematoxylin for 4 min and washed for 10 min with water. Differentiation was then performed in acid‐alcohol solution (1% hydrochloric acid in 70% ethanol) for 3 s, and cytoplasmic counterstaining was achieved using eosin Y for 25–60 s after washing. The treated slices were dehydrated through graded ethanol series (70%, 95%, and 100%), cleared in xylene, and coverslipped with permanent mounting medium (Thermo Fisher Scientific, 1900233).

The slides were scanned using the Hamamatsu Nanozoomer S360 digital pathology scanner at 40× magnification (0.46 μm/pixel resolution). The digital images were stored in a database and analyzed using dedicated image analysis software (NDP.view2 and Visiopharm) to assess the consistency between parental tumor tissue and GBOs.

### 2.7 Whole Exome Sequencing of GBOs for Different Status and Parental Tumors

The whole‐exome sequencing was conducted by Novogene Company. At least 2 pieces of parental tumor and 10–20 organoids including original GBOs, passaged GBOs, and recovered GBOs were snap‐frozen and stored in liquid nitrogen until processing. DNA was extracted and interrupted into fragments of 180–250 bp by a fragmentation device (Covaris). These library DNA were treated using end‐complement system and end‐addition poly(A) buffer reaction system and were ligated with Illumina sequencing adapters. Fragment size screening was performed using the Agencourt SPRIselect kit (Beckman Coulter, 2358413) and the original library was PCR‐amplified to ensure total volume. Exomeseq library was prepared using the SureSelect Human All Exon V6 kit/SureSelectXT Mouse All Exon library kit (Agilent) and was PCR‐amplified with 10–12 cycles. The completed exomeseq library was assessed using the Agilent 5400 system (AATI) and then sequenced on the Novaseq platform (Illumina) using Pair‐end 150 bp (PE150) sequencing.

The SNP and InDel sites were identified using SAMtools and counted. The exomeseq library was next analyzed with the known tumor somatic mutation database. The results were processed by using ComplexHeatmap, ggplot2, and Venndiagram to assess the consistency between organoids and parental tumor.

### 2.8 GBO Growth Analysis

The GBOs were cultured individually in a non‐TC treated 48‐well culture plate (Nest, 113023BK01) with 500‐μL culture medium per well to measure the growth of organoids. Growth of the GBOs was assessed with images taken by a brightfield microscope and HAYEAR software, respectively, every 3 days. The 2D area of each GBO was outlined and measured with ImageJ. The outlined area was divided by the original area to calculate a growth ratio, and the growth ratios for selected time points were analyzed by GraphPad to compare the growth curve of each GBO.

### 2.9 Drug Treatment of GBOs

To test drug susceptibility, the organoids from two patients were selected and cultured in individual wells of a 24‐well culture plate (Nest, 110623BH01) with 500 μL GBO medium containing either 160 μM temozolomide (MedChemExpress, HY‐17364) or DMSO vehicle control. To eliminate the influence of intra‐tumor heterogeneity, each treatment was triplicated, and the GBOs used in the experiment were selected with a diameter of 500 μm. The culture plates were placed on a shaker rotating in a sterile incubator to culture for weeks. The medium was replaced with fresh medium containing temozolomide or DMSO every 48 h; meanwhile, the 2D area of individual GBOs was outlined and measured. After 1 week, temozolomide and DMSO were withdrawn, and GBOs were kept culturing in culture medium for 2 weeks. The growth curve of the organoids was analyzed and compared with the results of patient follow‐up.

### 2.10 Immunofluorescence of Primary Glioblastoma Cells

Primary glioblastoma cells were cultured in a 24‐well plate with cell slides (Biosharp, BS‐09‐RC) for a week. The cell slides were washed with PBS and fixated with 4% paraformaldehyde (Servicebio, CR2210088) for 15 min at room temperature and then were washed with PBS and treated with PBST (0.5% Triton‐X100 in PBS) for 20 min. 5% BSA in PBS was used to block for 30 min. The BSA was dried and CD133 antibody (Thermo Fisher Scientific, 2324850) attenuated with donkey serum was dropped on the slides. The slides were incubated in a humid and dark box in 4°C overnight, and were washed with 0.5% PBST next day. Anti‐rabbit Alexa488‐conjucated antibody (Thermo Fisher Scientific, 53‐6503‐82) were dropped on slides and incubate at room temperature for 1 h. Washed slides with PBST for 3 times. 0.1% DAPI in PBS was added on the slides and incubated in a humid and dark box at room temperature for 10 min. Finally, the slides were sealed with antifade mounting medium (Biosharp, BL739B) and imaged by using fluorescence microscopy.

## 3 Results

### 3.1 Application of the Improved Method in Establishing the Organoid Model of Glioblastoma

Jacob et al. had reported a method to generate glioblastoma organoids from patient‐derived glioblastoma pieces [30]. The GBOs established with their method can keep the molecular heterogeneity, structure and microenvironment of glioblastoma. They also reported a method of biobanking, recovery and orthotopic transplantation in animals. However, their method requires a high demand for dissection skills, takes researchers more time to operate and asks for more experimental equipment. Many researchers could not successfully replicate the described organoid generation method and were compelled to generate organoids with structurally compromised microenvironment in Matrigel. Therefore, we improved their method and established 15 glioblastoma organoid models successfully from 18 patient‐derived glioblastoma samples. The improved method is easier and faster in operation, with a high success rate of 83% to generate organoids. Generally, a 5 mL tumor can be dissected within 2 h (Figure 1A). Despite the differences among samples from different patients that caused the variation of growth and metabolism, most of the organoids were formed in 1 to 2 weeks with consistent appearance (Figure 1B). The generated organoids can be passaged, stored, recovered and cultured for a long time.

**FIGURE 1:**
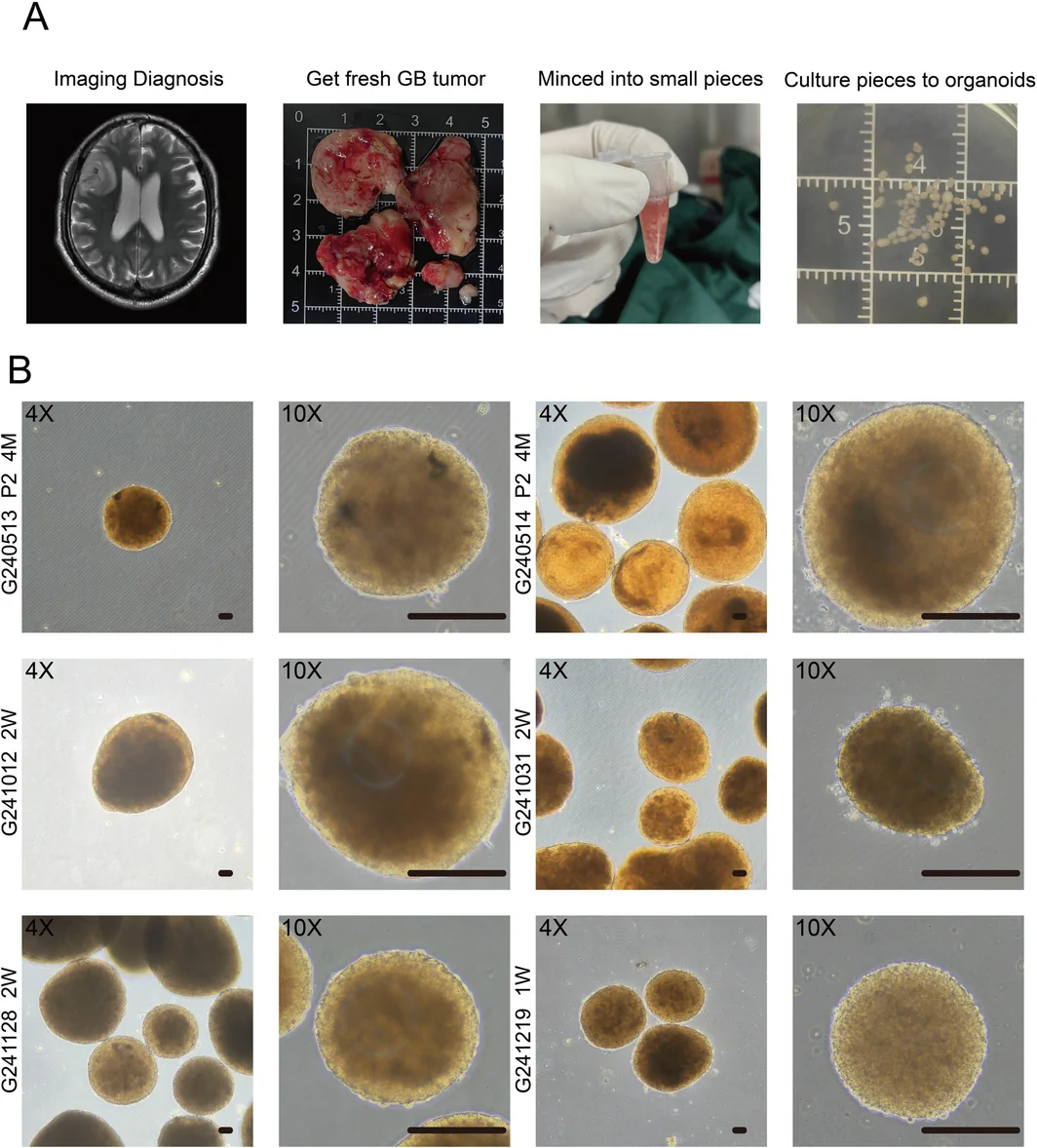
Generation GBOs from patient tissues with improved method. (A) A schematic of the procedure with MRI image and sample images. (B) Bright‐field images of GBOs from 6 patients with different appearances. Scale bar, 100 μm.

### 3.2 Maintenance of Structural and Molecular Intratumoral Heterogeneity by GBOs

To assess whether GBOs can maintain the structural and molecular characteristics of patient tumors, we performed H&E staining and whole‐exome sequencing for samples from the same patient, including tumor tissues, original organoids, passaged organoids, and recovered organoids. The GBOs displayed the nuclear atypia of patient tumors, containing abundant pleomorphic nuclei. These characteristics were maintained in the passaged GBOs and recovered GBOs (Figure 2A). We next performed the whole‐exome sequencing. The GBOs revealed an overlapped single nucleotide variants (SNVs) profile as 86.8% with the parental tumor tissue. Besides, the overlapped SNVs were found between the original organoids and passaged organoids (88.8%), which were also found between original organoids and recovered organoids (88.4%) (Figure 2B,D). The insertions and deletions (InDels) analysis was also performed among these samples, and the organoids for any status were found comprising a highly similar mutation expression with the parental tumor tissue (Figure 2C). These results revealed that GBOs largely maintain structural and molecular characteristics of parental tumors, including cellular distribution and genomic heterogeneity. Besides, these signatures were kept in the passaged and recovered organoids.

**FIGURE 2:**
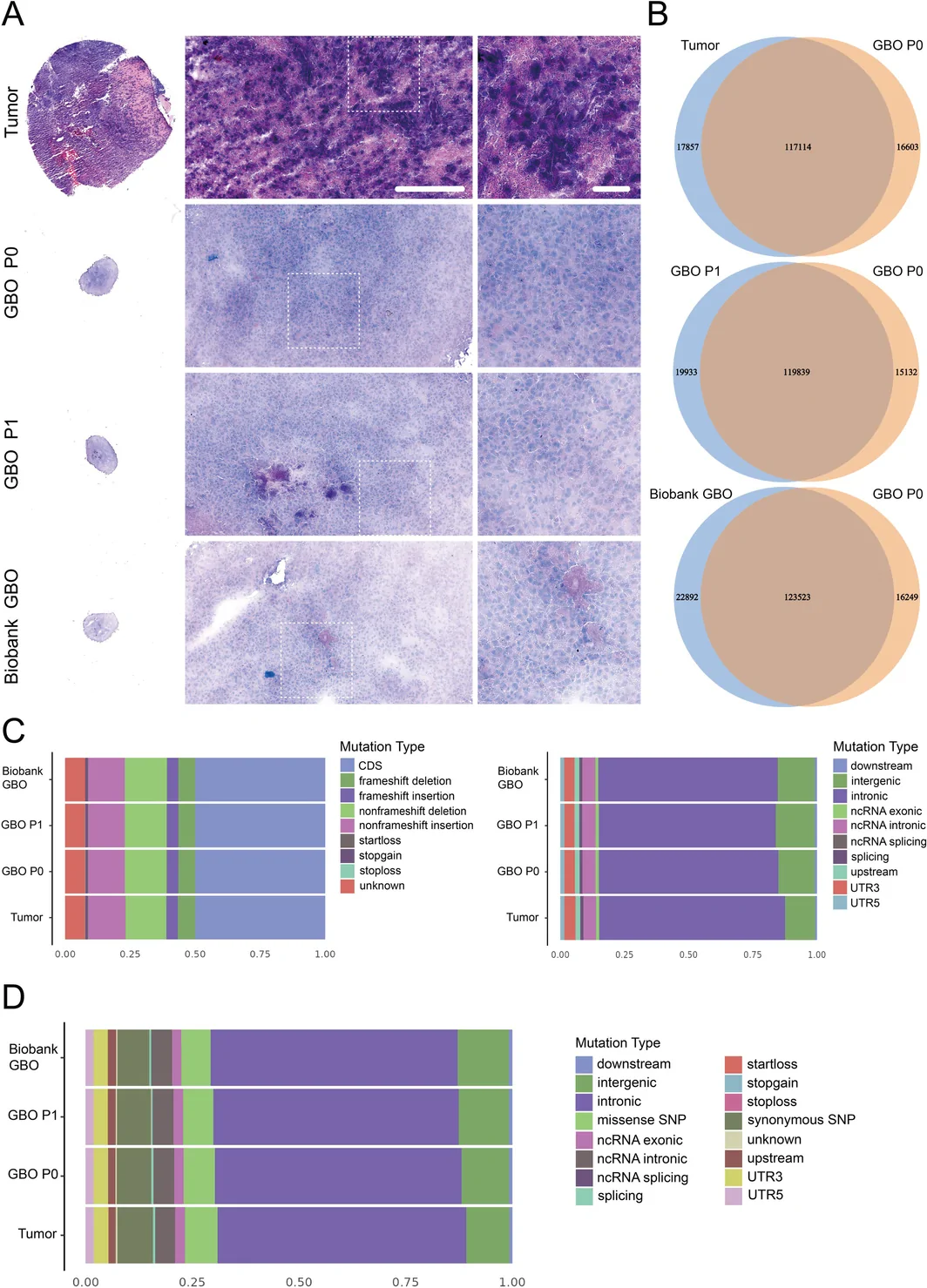
GBOs maintain histologic features and mutational profiles of parental tumors. (A) H&E staining images of parental tumor, corresponding GBO, passaged GBO and biobank GBO. Scale bars, 200 μm. (B) Venn diagram demonstrating 86.8% mutational (SNP) overlap between organoids and the parental tumor tissue, 88.8% mutational (SNP) overlap between the original organoids and the passaged organoids, and 88.4% mutational (SNP) overlap between the original organoids and the biobank organoids. (C) Proportion of the mutation types (SNP) detected in the parental tumor tissue and GBOs for different states. (D) Proportion of the mutation types (InDel) detected in the parental tumor tissue and GBOs for different states.

### 3.3 Isolation of Primary Glioblastoma Cells With the Improved Method of Generating Tumor Organoids

Despite the advantages of GBOs as an in vitro 3D model, the patient‐derived primary tumor cells are still widely used in experiments for co‐culture, observing cellular morphology and medicine testing [33]. We successfully isolated primary glioblastoma cells from the medium collected during generating GBOs (Figure 3A). The primary glioblastoma cells had different appearances (Figure 3B) and revealed the characteristics of the primary glioblastoma cells (CD133) [34] (Figure 3C). The primary cells isolated with our method had a higher positive rate of CD133 than those isolated with a traditional approach (Figure 3D). The isolated primary cells could be cultured for a long time and stored, which also makes up an important part of a resource for glioma research.

**FIGURE 3:**
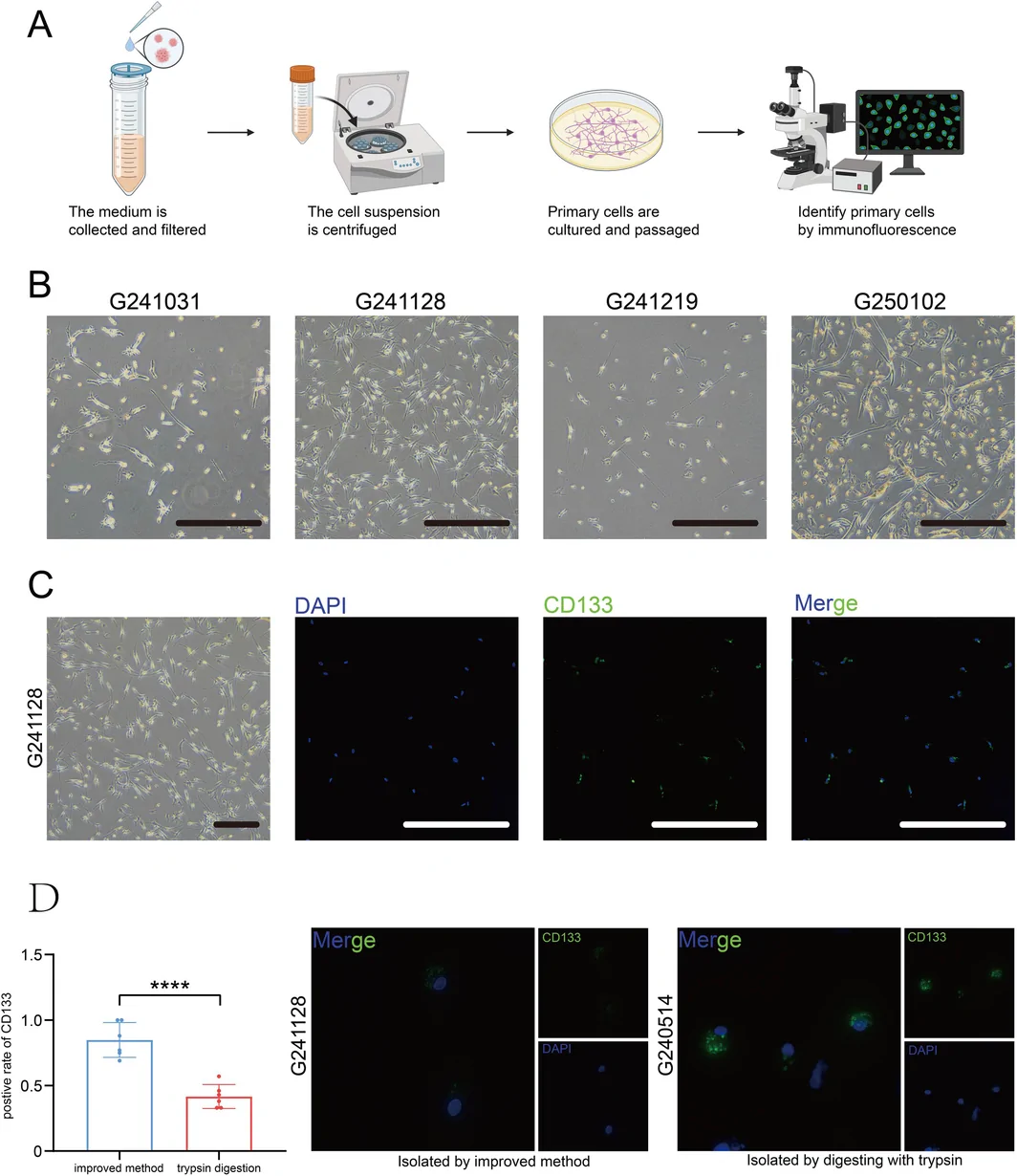
Primary glioblastoma cells from different patients. (A) Graphic abstract of isolating primary cells from tumors. (B) Bright‐field images of primary tumor cells from 4 patients with different appearances. Scale bar, 1 mm. (C) Bright‐field image and immunofluorescence images of primary tumor cell with proved expression of CD133. Scale bar, 500 μm. (D) Positive rate of CD133 of primary cells isolated by our method and digesting by trypsin. All data are expressed as mean ± standard deviation, *n* = 6/group, ****p < 0.0001.

### 3.4 Application of GBOs in Assessing Patient Sensitivity to Treatment and Providing Individual Treatment

Patients have different susceptibility to various clinical therapy, and empirical treatments sometimes are ineffective in improving prognosis [35]. GBOs have been proved as an appropriate tool for screening drug [36] and in vitro prediction [37] (Figure 4A). Referring to our previous result of effective concentration of temozolomide in U87 and A172 (Figure 4B), we treated the GBOs from two patients with culturing medium containing 160 μM temozolomide [1] (Figure 4C) and analyzed their growth curve (Figure 4D). We also conducted patient follow‐up to investigate their prognosis. We found that GBOs from different patients showed various drug susceptibility. We compared the results of in vitro experiments with the results of the patient follow‐up (Table 1). The patient (G231021) whose GBOs showed higher drug susceptibility (growth rate is 92% of the control group) appeared recurrence after using temozolomide for 1 year, while the patient (G230921) whose GBOs showed lower drug susceptibility (growth rate is 100% of the control group) was lost during the follow‐up. Drug susceptibility of the GBOs revealed in the in vitro experiment might be related to patients' susceptibility in clinical therapy.

**FIGURE 4:**
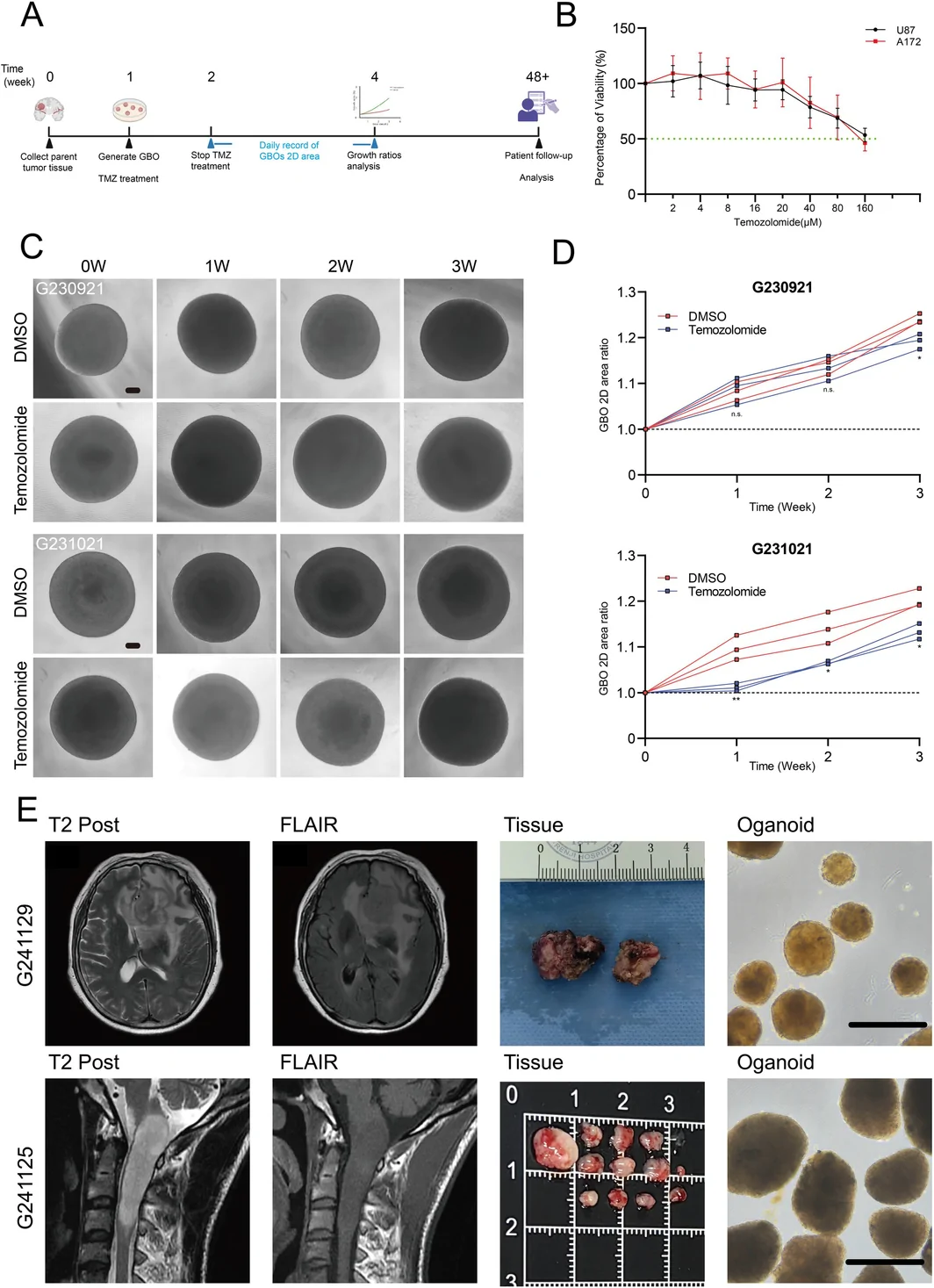
Application of the improved methods in CNS tumor organoids. (A) The schematic diagram of the drug experiment. G230921‐and G231021‐ parental tumor tissues were collected and the corresponding GBOs were generated in 1 week. The GBOs were next treated using DMSO and temozolomide for 1 week and were kept culturing for 2 weeks. The recorded 2D area of GBOs was analyzed after drug treatment. The prognosis of corresponding patients is fellow up after 1 year for analysis. (B) The glioblastoma cell lines were treated with temozolomide for 72 h and cell viability was detected by CCK8 assays. (C) Brightfield images of individual GBO growth over time following 3 weeks of exposure to drug treatment (160 μM temozolomide or DMSO) for G230921‐GBOs and G231021‐GBOs. Scale bars, 200 μm. (D) Quantification of relative GBO 2D area growth. Each curve represents individual GBO growth. Statistical significance noted on graph at same time points by *t*‐test with different colors indicating different treatments. (*n* = 3; *p < 0.01; **p < 0.01; n.s.: *p* > 0.05). (E) MRI T2 post‐contrast and FLAIR images of the patients (G241125, Subependymoma; G241129, Diffuse large B‐cell lymphoma). Images of tumor tissues and bright‐field images of organoids. Scale bars, 1 mm.

### 3.5 Generation Organoids From Other Primary CNS Tumor With Improved Method

As our improved method was confirmed to generate GBOs stably and efficiently, we collected tumor samples of subependymoma and diffuse large B‐cell lymphoma and generated their organoid models successfully (Figure 4E). The lymphoma organoids possessed small spherical structures, lower metabolism, and their size was smaller than GBO. The subependymoma organoids had irregular structures and smooth edges. Despite characteristics of other tumor organoids, such as volume, appearance and metabolism, are different from those of glioblastoma, they were also formed in 2 weeks and grew continuously under culture conditions.

## 4 Discussion

Glioblastoma has many unique biological characteristics, which make it one of the most malignant CNS tumors in adults. The prognosis of patients with glioblastoma is generally poor, and many current treatments fail to bring expected effects. Recent studies indicated that the unique microenvironment of glioblastoma is essential to the occurrence and development of the tumor. Therefore, establishing an in vitro model that can stimulate the glioblastoma microenvironment is critical to basic researches and clinical improvement.

Jacob et al. [30] reported a method to generate glioblastoma organoids maintaining heterogeneity in genetic characters and microenvironment of glioblastoma of the patients through mechanical fragmentation, which provides more real glioblastoma models. However, their method is hard to replicate due to its complicated requirements and high technical operation. The method requires a stereomicroscope for dissection, which may limit its application in many research institutes. The tumors are hard to mince into 0.5 to 1‐mm diameter pieces because of their movement in the medium. In most cases, researchers have to spend several hours to process tissues and generate a few organoids. The research field needs an improved method to promote glioblastoma organoids to become general in vitro models.

Several researches have been proceeded to improve the method and still have various deficiencies. Chun‐Chung Chen et al. [38] used the method in other brain tumors for the first time, but their method showed a low success rate and lacked enough samples of specific tumors; Anaïs Oudin et al. [39] reported a method to generate GBOs without using shakers. However, ignoring red blood cells would influence the growth of GBOs; Aurélie Soubéran et al. [40] recently reported a similar method with a requirement of a tissue chopper and had generated organoids of brain metastases, which might be more suitable for commercial use rather than experimental need; Kalil G. Abdullah et al. [41] made first use of the method to generate low‐grade glioma, and their result encourages us in our work about generating low‐grade glioma organoids from a cervical cord tumor; Tianping Peng et al. [42] also reported a new method, but the co‐culture system of tumoroids and brain organoids was harder to operate and popularize in many areas.

Compared with these methods, our method is easier and faster in operation, with a high success rate, and independent from some special equipment. Therefore, the improved method can generate organoids from more tumor samples in limited time. Moreover, our report proves that spinal tumors can be collected to generate tumoroids through the improved methods as brain tumors. Above all, it's the first reported method that allows researchers to isolate primary tumor cells from the collected medium during the establishment of GBOs simultaneously. The primary glioblastoma cells can be used in co‐culture and to test drug susceptibility, providing important support for organoid‐based experiments. The paired in vitro models derived from the same patient provide more convincing evidence to support the hypothesis. Even though the significance of organoids has been emphasized, patient‐derived tumor xenograft models remain an indispensable component in pharmacokinetic research. Therefore, our method allows us to establish 3D (organoids) and 2D (primary cells) models from the same tumor sample economically, efficiently, and simultaneously. The organoid model has potential in assessing the success rate in clinical transformation of basic research and predicting clinical trials (Figure 5). These advantages will promote the application of GBOs in research. It is worth noting that organoids were difficult to generate from recurrent glioblastoma samples following chemoradiotherapy. We assume this phenomenon might be associated with the reduction of tumor stem cells after chemoradiotherapy and are exploring the method to generate organoids from recurrent glioblastomas. On the other hand, our research reports a method for the isolation and short‐term culture of primary tumor cells. However, suspension culture is a better choice for long‐term culture, as adherent culture has been proved as a negative factor for the maintenance of cell stemness.

**FIGURE 5:**
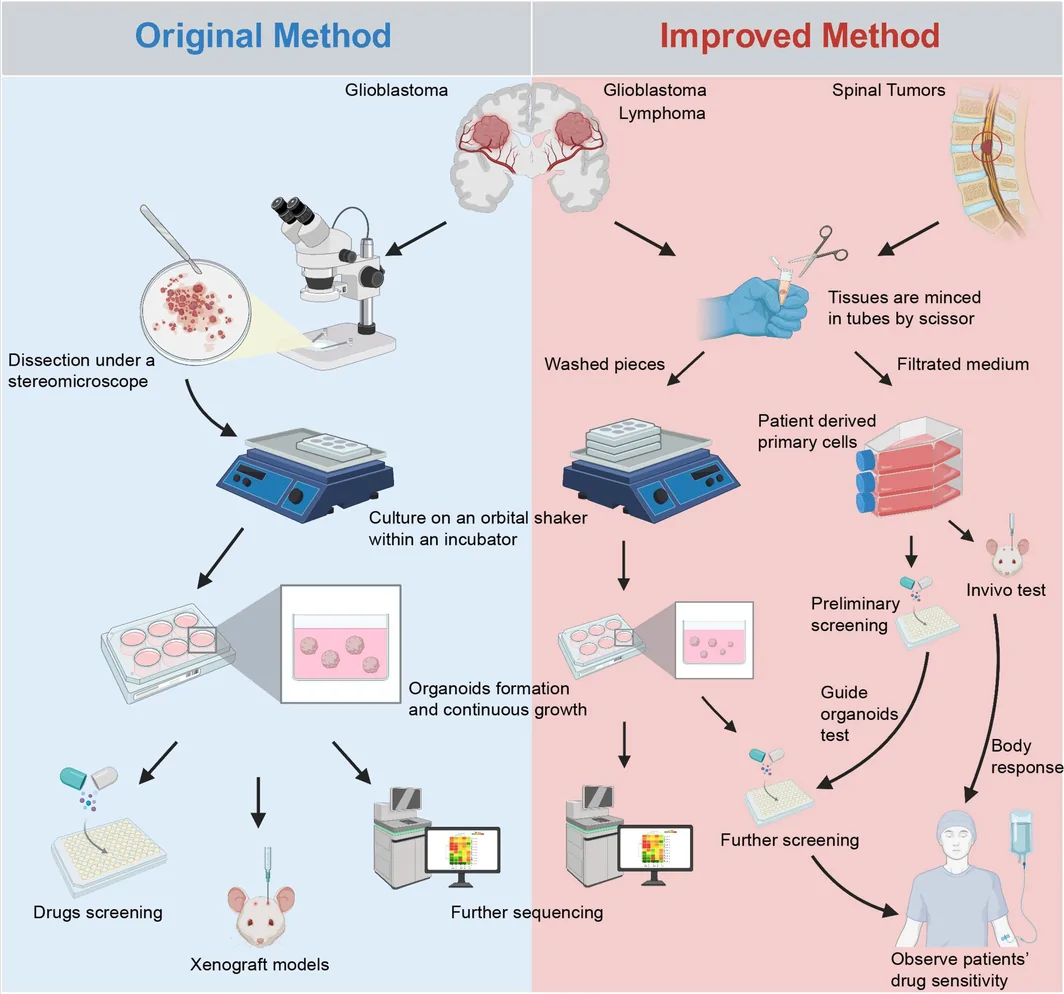
A comparison between the original method and the improved method. This figure shows the differences between two methods. The improved method expands the range of tumor types that can be treated. Organoids and primary cells are derived from the same sample. The primary cells are used for drug screening and PDX models establishment. These results will support the organoid experiments and collectively guide the clinical treatment decisions.

The current standard of care therapy of glioblastoma is maximal surgical resection followed by chemoradiotherapy, and the therapeutic efficacy is assessed by regular follow‐up after operation. The prognosis of clinical treatments is unpredictable due to heterogeneity among the patients. Clinical trials on novel glioblastoma therapeutic methods are always time‐consuming and generally fail to obtain the expected results. In this paper, we conducted an in vitro experiment of temozolomide susceptibility with the GBOs from different patients. The results were compared with the results of patient follow‐up to predict therapeutic efficacy of temozolomide. Even though both patients show non‐methylation for the status of MGMT and both of their results of the follow‐up reveal bad prognosis, the patients still have different disease processes. We have proved that our GBO might be an ideal in vitro model that can simulate patients' situations to a large extent and has the same susceptibility as the organoid generated from tumor stem cells [43]. Therefore, the GBOs could be used potentially in suggesting individual therapies to patients. The application of organoids can raise the success rate of translating basic research to clinical application and is efficient to predict the results of clinical trials. In the other hand, capillary structure of organoids is important to stimulate microenvironments and drug‐infiltrating [44, 45]. However, few vessels formed and distributed in GBOs, resulting in the action of drugs not reaching the center of the organoids. Therefore, we are trying to isolate vascular endothelial cells and working on a vascularized glioblastoma organoid model in future research. Furthermore, the current application of tumoroids was focused on chemotherapy, and treatments including radiotherapy and tumor‐treating field (TTF), which were important parts of clinical therapy, were hard to conduct in labs. New devices were required to meet these needs. We were working on a microfluidic chip that can treat individual GBO with TTF in an incubator. We hoped that our work will imitate the current standard of clinical therapy in the future and provide more reliable advice for individual treatment.

Besides glioblastoma, there are many other kinds of primary tumors in the central nervous system, such as lymphoma and metastatic tumors, etc. Patients with CNS tumors always have poor prognosis and high fatality rates caused by widely intracranial diffusion, serious encephal edema, and cerebral hernia. Up to date, few organoid models of CNS tumors except glioblastoma are established. In our study, we generated organoids of subependymomas and diffuse large B‐cell lymphoma from tumor samples of patients successfully with our improved method. The organoids of subependymomas and diffuse large B‐cell lymphoma exhibited many similarities to glioblastoma organoids, and many differences in growth rate and metabolic capability. Compared with GBOs, diffuse large B‐cell lymphoma organoids were small spheres with a diameter of 200 to 300 μm and couldn't be cultured in GBO culture medium, where it was observed that organoids maintain structure and can't keep growing. The organoids of subependymomas were smooth and irregular tumoroids with a diameter of 500 μm, and the organoids tended to present with central necrosis earlier in the growth. These results proved that our improved method might be used to generate organoid models for other CNS tumors except glioblastoma, which will promote the development of basic research on nervous system tumors. We suppose the high‐water content of brain tissue causes the consistency of the method for generating organoids from pieces of various brain tumors in liquid medium. Despite the method for generating tumoroids being similar, the process and conditions of culturing should be adjusted for the types of brain tumors. We will keep working to try and summarize the methods of generating other CNS tumors, ultimately establishing a resource for basic and translational research of CNS tumors.

## 5 Conclusion

Commencing with surgical samples from glioblastoma patients, we report an improved method to generate glioblastoma organoids simultaneously, easily, rapidly, and economically. More organoids can be generated in less time with a high success rate in this way, and primary tumor cells can be isolated at the same time. We also conducted in vitro experiments with glioblastoma organoids and assessed the potential applicability of glioblastoma organoids for predicting patients' prognosis, suggesting postoperative treatments, and finding suitable experimental subjects for clinical trials. Finally, we successfully generated organoids of subependymomas and diffuse large B‐cell lymphoma from two patients' tumor samples with an improved method. This study provides a simplified method to generate organoids of CNS tumors as an authentic in vitro model. Researchers of CNS tumors will benefit from the improvement of the method, and patients will benefit from the development of medical research.

## Author Contributions

S.W. and Z.S. performed the experiments and the statistical analysis, C.Z. performed the patient follow‐up. Y.M., D.D., X.Y., and Z.Z. performed the operations and provided tissue samples. S.W. designed the experiments and wrote the manuscript. Y.Q. supervised the project. Y.Q. and Y.M. provided comments to the project. Q.Q. and W.S. gave help to the writing. All the authors reviewed and accepted the contents of the article.

## Funding

This work was supported by grants from National Natural Science Foundation of China (82272728), Shanghai Association for Science and Technology (201409002400), and Minhang Municipal Commission of Health (2024MZYS26).

## Ethics Statement

Glioma tissues and other central nervous system tumor samples were in accordance with the ethical guidelines of Ren Ji Hospital, School of Medicine, Shanghai Jiao Tong University (IRB number, RA‐2022‐185).

## Conflicts of Interest

The authors declare no conflicts of interest.

## Data Availability

The data that support the findings of this study are available from the corresponding author upon reasonable request.
